# Perceptions of and challenges faced by primary healthcare workers about preconception services in rural India: A qualitative study using focus group discussion

**DOI:** 10.3389/fpubh.2022.888708

**Published:** 2022-08-17

**Authors:** Amruta Paresh Chutke, Prakash Prabhakarrao Doke, Jayashree Sachin Gothankar, Prasad Dnyandeo Pore, Sonali Hemant Palkar, Archana Vasantrao Patil, Aniruddha Vinayakrao Deshpande, Khanindra Kumar Bhuyan, Madhusudan Vaman Karnataki, Aparna Nishikant Shrotri

**Affiliations:** ^1^Department of Community Medicine, Bharati Vidyapeeth Deemed University Medical College, Pune, India; ^2^State Family Welfare Bureau, Department of Public Health, Government of Maharashtra, Pune, India; ^3^United Nations Children's Funds (UNICEF), Mumbai, India

**Keywords:** preconception care, qualitative research, focus group discussion, Socio-Ecological Model, healthcare workers, challenges, suggestions

## Abstract

**Background:**

Providing preconception care through healthcare workers at the primary health care level is a crucial intervention to reduce adverse pregnancy outcomes, consequently reducing neonatal mortality. Despite the availability of evidence, this window of opportunity remains unaddressed in many countries, including India. The public health care system is primarily accessed by rural and tribal Indian population. It is essential to know the frontline healthcare workers perception about preconception care. The study aimed to identify barriers and suggestions for framing appropriate strategies for implementing preconception care through primary health centers.

**Methods:**

The authors conducted a qualitative study using focus group discussions (FGDs) with 45 healthcare workers in four FGDs (8–14 participants in each), in four blocks of Nashik district. The transcribed discussions were analyzed in MAXQDA software using the Socio-Ecological Model as an initial coding guide, including four levels of factors (individual, interpersonal, community, and institutional) that influenced an individual's behavior to use preconception care services.

**Results:**

Healthcare workers had some knowledge about preconception care, limited to adolescent health and family planning services. The interpersonal factors included heavy workload, stress, lack of support and co-operation, and paucity of appreciation, and motivation. The perceived community factors included poverty, migration, poor knowledge of preconception care, lack of felt need for preconception services, the influence of older women in the household decision, low male involvement, myths and misconceptions regarding preconception services. The identified institutional factors were lack of human resources, specialized services, logistics, and challenges in delivering adolescent health and family planning programs. Healthcare workers suggested the need for program-specific guidelines, training and capacity building of human resources, an un-interrupted supply of logistics, and a unique community awareness drive supporting preconception care services.

**Conclusion:**

Multi-level factors of the Socio-Ecological Model influencing the preconception care services should be considered for framing strategies in the implementation of comprehensive preconception care as a part of a continuum of care for life cycle phases of women.

## Introduction

There has been increasing focus on Preconception care (PCC) globally, mainly due to its contribution to reducing adverse pregnancy outcomes. The present continuum of care does not include care before pregnancy. PCC bridges the gap in the continuum of care and aims to reduce parental risk factors before conception, thereby improving maternal and infant outcomes (1). The effectiveness of preconception interventions has been documented since 1979 (2). The systematic reviews since 2002 have strengthened the evidence of the utility of preconception interventions in reducing adverse pregnancy outcomes even in Low- and Middle-Income Countries (LMICs) (3–10). Despite the availability of evidence-based PCC interventions, only Bangladesh (11), Philippines, Sri Lanka (12), China (13), United States of America (USA) (6), and United Kingdom (UK) (14) have provided guidelines and introduced PCC in their public health services (15). This window of opportunity for the timely achievement of Sustainable Development Goals (SDGs) remains unaddressed for most countries, including India.

The maternal mortality ratio for India in the twenty-first century has declined from 301 (2001–2003) to 113 per 100,000 live birth (2016–18) (16). Similarly, the under-five child mortality rate has considerably reduced from 59 (2010) to 36 (2018) in the last decade (17). Nevertheless, the pace of this reduction has been slowed down in recent years (18, 19). World Health Organization (WHO) has recommended the rollout of PCC activities globally in 2013 (12). The government of India also included PCC in the India Newborn Action Plan, affirming PCC as one of the most critical pillars of the intervention package to address stillbirth and newborn health (20). The Federation of Obstetric and Gynecological Societies (FOGSI), India, circulated good clinical practices on PCC (21). However, these recommendations remain limited to ‘specialists' groups, extending services to upper socio-economic classes only. The primary health service provider in rural areas is the public sector (72%) (22). In India, presently PCC concept is limited to birth spacing services. To reach the rural, especially the vulnerable tribal population, and to widen the scope of PCC, implementing PCC services through the public health system in India is necessary. It is inevitable to consider the knowledge and perceptions of health workers to have a robust strategy.

PCC involves identifying and modifying potential risks for adverse pregnancy outcomes in the preconception period. Studies have suggested that identifying practical and feasible interventions based on local situations are crucial (23–26). Some health service research studies have identified factors contributing to the delivery and uptake of the PCC. Most of these studies are from High-Income Countries (27–36), and very few from Low-Income Countries (37–39). Studies from High-Income Countries identified lack of knowledge, awareness, demand, planning, publicity, unclear division of responsibilities, and poor coordination as challenges perceived by healthcare providers in providing PCC services (29, 31, 33, 35, 40). Health behavior of an individual is determined by several factors and it is important to know the knowledge and perceptions of the frontline healthcare workers (HCW). HCW are the first contact health care providers in the community and have a pivotal role in providing services. The extent of knowledge, behavior, and perception about PCC among HCW in India is almost unknown except for adolescent healthcare services and family planning services. To our knowledge, this is the first qualitative study in India that seeks the perceptions of frontline healthcare workers about preconception care. The study aims to explore the healthcare workers knowledge and perceptions about challenges and strategies in introducing PCC. This study is a part of a comprehensive intervention study that broadly aims to reduce low birth weight and preterm births by addressing the known risk factors in the preconception period. The knowledge and behavior among women desiring pregnancy regarding preconception care are reported elsewhere (41).

## Methods

The present paper uses the consolidated criteria for reporting qualitative research (COREQ) guidelines.

### Study design

This is a qualitative study using Focus Group Discussions (FGD).

### Study setting

**Figure 1:**
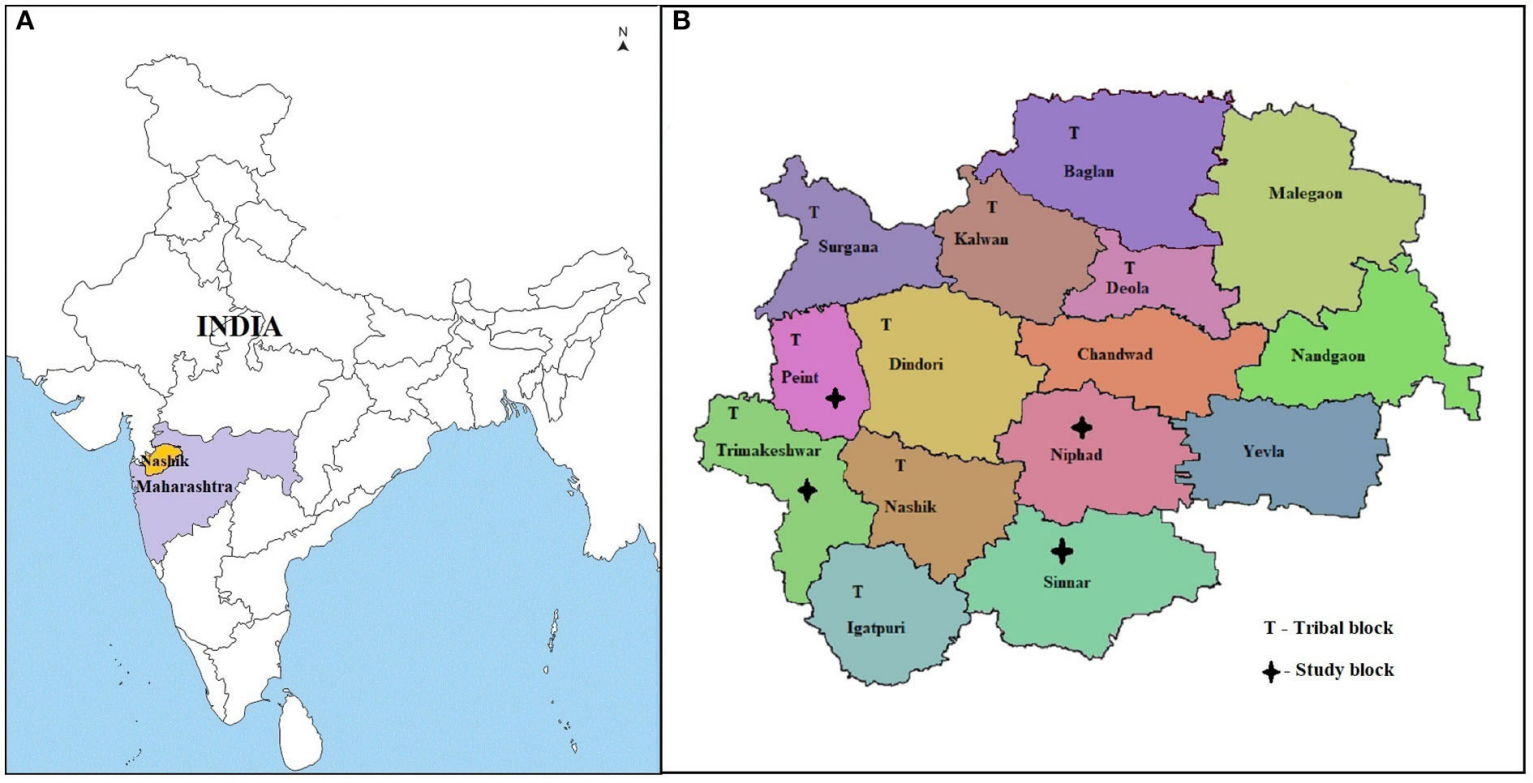
Map of India showing the location of the study area, 2018-19. **(A)** India showing the location of selected Nashik district, State Maharashtra **(B)** Nashik district with selected study blocks. Source: Maps of India freely available and accessible at www.mapsofindia.com.

The authors conducted the study in rural areas of Nashik District, Maharashtra, India, covering a population of 6,109,052 (22). Nashik district was selected purposively as the district is a tribal dominant district. There are 15 blocks in the district, of which the government had notified nine blocks as tribal. Four blocks were selected, of which one tribal block and one non-tribal block were selected randomly as intervention blocks. Furthermore, adjacent one tribal and one non-tribal block were selected to match socio-cultural and geographical features as comparison blocks. Thus, two tribal and two non-tribal blocks were selected, covering a population of 976,149 individuals (15.98% of the district population). Figure 1 shows the locations of selected blocks.

Table 1 gives the demographic information of selected blocks as per Census 2011 (22).

**Table 1:** Demographic information of study area, Nashik as per Census 2011, India.

**Block**	**Population as per census**	**Area (km^2^)**	**Pop. Density**	**PHCs**	**No. of villages**	**No. of ANM**	**No. of MPW**
Sinnar (NT)	281,091	1,343.79	258	6	129	40	21
Peint (T)	119,838	556.64	215	7	144	42	14
Niphad (NT)	418,853	1,048.63	470	9	134	42	28
Trimbakeshwar (T)	156,367	900.27	187	6	125	40	24

*T, Tribal; NT, Non tribal; PHC, Primary Health Center; ANM, Auxiliary Nurse Midwife; MPW, Multi-Purpose Worker*.

The public healthcare system in rural and tribal India is mainly through the Primary Health Center and Health Sub-Centers. Nashik district has 108 Primary Health Centers (PHCs) (each covering about 20,000–30,000 population each and having about 15 healthcare workers) and 592 Health Sub-Centers (HSC) (each covering about 3,000–5,000 population and having three healthcare workers).

### Participants and recruitment

Female health workers (Auxiliary Nurse Midwife- ANM) and male healthcare workers (Multi-Purpose Workers- MPW) were the study participants working primarily at PHC and HSCs, and some of which were contractual. The MPW and ANM receive 1 year, and one and a half years of special professional health care training, respectively, after passing 10 years of regular schooling. About one-third of health workers [83] from study blocks were approached and informed through the institutional head about the purpose of the study, date, and venue of the FGD. A total of 45 HCW who volunteered to participate in four FGDs were recruited through convenient sampling. The team had planned to conduct one FGD in each block, and accordingly four FGDs were conducted. The authors had not planned to conduct FGDs till the data saturation point.

### Data collection

An interview guide was used as a tool for data collection and it was prepared through a consultative process. The interview guide was validated, translated into the local language (Marathi), back-translated (English), and pre-tested in another rural area with similar settings. The interview guide consisted of domains, sub-domains, and probes. The probes were open-ended to allow the HCW to describe their knowledge, perception, and practices; identify challenges and possible strategies to deliver PCC services (Supplementary File S1).

All FGDs were conducted in June 2018 by four investigators, who are teaching faculties from a medical college and assisted by two research coordinators. Out of six members, four are females. All faculties are post-graduate in community medicine, one of them is doctorate also, and the research coordinators are post-graduate in public health. All the members are trained in conducting qualitative research and FGDs and have 5–42 years experience of doing research. The faculties conducted FGDs, and the coordinators facilitated by taking notes. FGDs were conducted in a separate room in the PHC, with exclusive presence of research team and participants only. Each research team conducting a FGD had two members, one faculty and one research coordinator. The authors ensured representation from all the PHC of the selected blocks. Before starting FGD, written informed consent was obtained from the HCW for participation, audio recordings, and subsequent publication. The facilitator ensured that the moderators covered all domains during discussions. The information about participant's age, sex, education, work-station, and work experience was collected. The facilitator gave the participants numbered placards, and the moderator addressed them by their respective placard numbers during FGD. They were also encouraged to freely discuss their perceptions, views, and experiences. All FGDs were conducted in the local language (Marathi). The research team and participants met for the first time during the FGD. The moderators first briefly introduced the project and further discussed domain and sub-domains. The moderator used probes if the discussion got diverted from the topic. Each FGD was conducted for about 50 minutes.

### Data analysis

For accuracy, all FGDs were transcribed verbatim and checked several times against recordings and notes. Data were analyzed using a content analysis deductive approach using the Socio-Ecological Model (SEM) (42). The SEM is a theory-based framework to understand the multi-level and interactive effect of personal and environmental factors that determine the behavior of an individual and is used in many qualitative research studies. The framework includes factors at individual level, interpersonal level, i.e., among peers and colleagues, community and social level, and institutional levels, i.e., medical and social services (43). The codes namely individual, interpersonal, community and social, and institutional levels were used as an initial coding guide to describe themes. Newly derived codes representing each of these themes were added to the framework to build our model of factors influencing PCC. Data were analyzed using MAXQDA version 20.2. Three researchers, who were part of conducting FGD independently identified themes and sub-themes and finalized them through discussion with the remaining research team members. Any disagreement in themes was discussed and resolved. The research team translated the transcripts into English and included the unique quotes in Results Section. The data is deposited in the department.

## Results

Table 2 gives the details of the participants. Forty-five health care providers (31 female and 14 males) participated in these four FGDs, with 8–14 participants in each group.

**Table 2:** Demographic characteristics of focus group discussion participants, Nashik, India 2018–19 (*n* = 45).

**Demographic characteristics**	**FGD 1**	**FGD 2**	**FGD 3**	**FGD 4**
Participant number	14	11	8	12
Age in years mean (SD)	39.00 (7.26)	38.00 (8.72)	42.50 (12.24)	47.42 (6.42)
Years of education mean (SD)	11.71 (1.38)	11.45 (2.42)	12.63 (2.72)	10.33 (0.78)
Years of experience mean (SD)	15.50 (6.20)	16.27 (10.35)	13.38 (7.63)	23.33 (9.90)

*FGD, Focus group discussion*.

Table 3 gives the identified themes and sub-themes under four levels of SEM including the unique quotes denoting the participant and focus group numbers. Identified sub-themes were almost similar for tribal and non-tribal blocks; hence combined results are presented.

**Table 3:** Identified themes and sub-theme based on Socio-Ecological Model after focus group discussion.

**SEM levels**	**Themes**	**Sub-themes**	**Aspects**	**Quotes**
Individual	Knowledge of health care workers	Components	Physical and mental health of women	P5, G3: Providing them (newly married women) services to increase the hemoglobin level and to make these women ready for pregnancy P4, G4: Preconception care is needed to create a healthy mother to be able to conceive a healthy child. P7, G1: Not only physically, but the woman should be mentally prepared for pregnancy. She has to take responsibility for the future baby. P7, G2: It is important to identify high-risk women before she conceives.
			Existing health programs	P1, G2: We provide iron tablets to adolescent girls through the Weekly Iron and Folic acid (WIFS) program. P4, G1: We provide folic acid tables for married women, advise them on an appropriate diet. We provide family planning services if a woman desires to delay her pregnancy after a baby's birth.
Interpersonal	Work environment	Workload	Work difficulties	P6, G3: The home visits are reduced due to the online workload. Authorities ask us to submit online data of different health indicators too frequently. Hence, it hampers our daily activities in terms of quality and quantity. We are expected to submit everything online and timely. P6, G3: PHC has electricity but a poor network, and when the internet works, there may not be an electric supply. Our time passes in vain without any work or any activity. We keep getting messages from Taluka Office to send the reports. For the last 2 days, there is no electricity in ….village.
			Stress	P4, G3: There is too much stress and tension, especially in reporting. Everyone is going behind reports and numbers in this digitalization of India. The health of people working in the health department only is being affected due to this work stress.
		Support and co-operation	Lack of support and co-operation	P7, G1: ANM and AWW are constantly in touch with the community. Their support is essential, and at the same time, seniors should guide adequately. P4, G3: AWW and ASHA have conflicts among themselves at times. In that case, they do not cooperate with each other and even with us. AWW does not take any responsibility in that case.
			Paucity of appreciation and motivation	P7, G3: A health worker has to walk a long distances in the interior of villages to do home visits, but it is not valued. Instead, sitting at PHC office and online timely submitted work is rewarded. Seniors should appreciate and support our field work.
Community and social	Socio-economic	Poverty	Poor access to health services	P12, G4: The household situation of people here are so poor that the girl takes home the supplementary food given in schools. She does not eat the food alone but shares it with the entire household members.
		Migration	Poor access to health services	P1, G2: People here are poor. So, the newly married couple migrates to earn livelihood for a more extended period. They return after 5 or 6 months when already conception has occurred. They rarely access services during this period and register ANC only after they are back.
	Socio-cultural norms and practices	Knowledge of community	Meager knowledge	P3, G3: Most women become pregnant immediately after marriage, and they do not know anything about pre-pregnancy care or need of pre-pregnancy care. P4, G4: Even if we try to get the women for a health check-up before pregnancy, the household members do not allow it. They shout at us.
			Paucity of planning pregnancy	P10, G1: If we ask them whether they want to conceive, then they become speechless. They never think of conception by themselves.
			Low acceptance of family planning services	P8, G2: Couples and families want a child immediately after marriage, within 1 year. The second pregnancy is also within 7–8 months of first delivery. There is no concept of using family planning services or contraceptives among women.
			Adolescent pregnancies	P1, G4: We have been working for many years. We have seen girls of age 12–14 years, and being pregnant. They do not know anything about pre-pregnancy care. P5, G3: People get their girls (daughters) married as soon as they complete education till 10th, the girls are generally just 16 or 17 years old. These girls are expected to conceive immediately after marriage.
		Felt need	Lack of felt need	P4, G3: Women do not disclose even if they are pregnant till the first 3 months. Therefore, they reach late for registering ANC.
			Non-consumption of balanced diet	P4, G3: In town, they (women) have breakfast, lunch, dinner, eat salad, etc. On the contrary, in villages, most women eat in the morning and leave their house for work. After returning, they have dinner. P2, G3: Some women are almost in the kitchen for 24 hours; even then, they remain undernourished and anemic.
		Substance abuse	Non-acceptance of suggestion	P5, G4: Few women consume tobacco and alcohol even during pregnancy, and if we ask them to stop, then their husbands think that we are interfering too much in their life. Few listen to us, but few do not cooperate.
		Influence of older women	Higher decision autonomy with older women	P4, G1: The decision of pregnancy of the daughter-in-law in a household lies with the older women of the household. Moreover, it is desired by the mother-in-law and the entire family that the newly married woman conceives soon after the marriage. P10, G1: Woman (younger ones) herself does not make decision
			Low acceptance of felt need	P7, G1: If the first child is girl, then they quickly plan for pregnancy. But if it is a male child then they prefer not to conceive soon. Women seek help only in such case when they do not want a pregnancy.
			Resistance to planning pregnancy by daughter-in-law	P4, G1: Mother-in-law does not allow her daughter-in-law or son to delay pregnancy, especially after the marriage of after first girl child. P14, G1: If we give any medications of IFA to the younger women before pregnancy, the older women ask, “Why are you giving these tablets? She is not pregnant and not having any problem, then why? If she conceives, the baby will grow more and become fat due to these tablets.”
			Resistance to use of family planning methods by daughter-in-law	P3, G3: We refer women to a civil hospital to get IUCD inserted after delivery. They go there and get it done. Few of them experience problems with Copper-T, so they go to a private hospital and remove it. Few also remove due to myths about use of Copper-T. Later, these women do not use any other contraceptive and again come to register for ANC with poor health conditions and anemia.
			Gender bias	P10, G1: Most importantly, they should know that a woman is not responsible for the gender of a child, and a child is the responsibility of both parents. P8, G4: Thorough examination and investigation should be done of a couple before pregnancy. A woman is ready for testing, but usually, the man is not. He is afraid of any problem found in him… In-laws are not ready to leave their biased thoughts…
		Role of men	Indecisiveness to child-rearing responsibility	P3, G3: Sometimes, even the men are young and cannot take the responsibility of being a father. They do not know what to do if his wife is pregnant.
			Poor acceptance of FP methods use	P14, G1: Males generally are not counseled for using contraceptives. Generally, family and community members do not support contraceptive use by males. We do not talk about contraceptives with men who are recently married.
		Myths and misconceptions	Myths about practices	P8, G3: A woman is not allowed to eat groundnuts, banana, papaya, or curd before conception. Also, she is not allowed to eat outside food. People think that this may cause abortion.
				P4, G4: About 20–25% of pregnant women do not reveal pregnancy in first trimester. People think that if they reveal their pregnancy, this may cause miscarriage or they may have to face pregnancy complications. Sometimes, these women reveal pregnancy in fifth month
			Myths about medicines provided	P6, G3: People do not value things provided free of costs, such as iron and folic acid tablets.
			Misconceptions about quality of health services	P6, G4: People think that the services provided in government hospitals are not good quality as the services are freely available. But it is not so. If people are charged highly for the services, then think they are good. P8, G2: We did not get any case of sexually transmitted diseases. People are superstitious. They seek the help of any traditional person (Baba) but they will not come to us. Even if there is abortion, they don't come to us.
Organizational	Resources	Workforce	Scarce human resources	P2, G3: Manpower is less. There are many vacant posts.
			Disparities in remuneration	P5, G4: Why are there differences in contractual and permanent staff payment? Payment for contractual staff is very low and they work same as a permanent staff.
		Specialized services	Lack of specialized services	P3, G3: On every 9th day of the month, an ANC camp is organized at a PHC in Nashik under…. scheme. Nearly 70–80 women come. Many times, there is no gynecologist available for check-up.
		Logistics	Lack of diagnostic kits	P6, G4: We go for field visits and sometimes, we do not have test kits or strips. Then people say that why have you come to visit when you do not have anything.
			Lack of medicine supplies	P8, G3: We used to get medicines at sub-center level. But now we get money…. And we have to buy everything from this amount including stationary, internet cafe for online reporting etc. This amount is not enough.
	Health services	Adolescent health services	School drop-outs and migration	P5, G2: We provide Iron and folic acid tablets to adolescent girls below 18 years. The girls who go to school receive the tablets. Migrant girls are left. In each school, about 5 to 6 girls migrate. P5, G3: We provide tablets to teachers, and teachers give them to the school girls. However, while distributing, they are apprehensive that complications may occur. So at times, there is non-compliance from teachers.
		Family planning and other services	Sub-optimal referral system	P4, G3: If any chronic condition is detected, we refer people to the higher center. But, at the higher center, people do not get proper services. P2, G3: When we go in the field, then, people say that the health staff at the referred center do not give us good services. P5, G3: People say that we should get services where the test is conducted and diagnosis is done, not refer elsewhere.
			Ill-effect of monetary incentives	P5, G3: Recently a scheme called….has become popular, people know that they get…. Rupees, so women come within 12 weeks of gestation. ANC Registration has increased because of this scheme. People come when they are given the temptation of something. People will come for money and not for the healthy baby.
Suggestions	Strengthening health systems	Workforce	Provision of specific guidelines	P1, G2: We provide health services to adolescent girls and married women like iron and folic acid supplementation or provision of birth spacing methods. However, these are mainly during pregnancy. There are no specific guidelines for services before pregnancy. Those should be provided.
			Increase in human resource	P4, G3: Firstly, all vacant posts should be filled
			Reduce disparities in remuneration	P4, G3: Remuneration of contractual staff should be increased.
			Capacity building and training	P3, G4: All health care staff should be well trained and re-trained to provide these pre-pregnancy services
			Support and co-operation	P2, G4: ASHAs and AWW have a lesser population to cater services, and they stay in the community. They both should support and co-operate with each other and with us to provide services to the community.
			Proper allocation of responsibilities	P4, G3: Increase the staffing and then increase our work. This work should be distributed appropriately among all health staff.
			Availability of specialized services at PHC	P7, G3: …. If their (community) problems are solved at the PHC level by specialist doctors, their trust in the PHC will increase.
		Infrastructure	Availability of diagnostic and medicine supplies	P8, G4: Diagnostic kits and medicines are needed and should be available. Earlier, we used to get the medicines at sub-center. But now, they give money…. We have to fit medicines, stationery, online reporting, etc. everything in this amount, and that is difficult. P6, G4: We go in the field to provide services. But if medicines are not there, people say, why are you here when you do not have any medicines.
			Strong referral system	P5, G3: If we refer to RH, then, at least the people there should treat the patient properly. Otherwise, people do not cooperate with us during our subsequent field visit. They ignore us.
		Special PCC program	Adolescent health program	P2, G3: Awareness should be created among girls and boys, and they should be given health education about preconception care in the schools. P5, G3: Intervention at adolescent age is necessary. Introduction to their own body, sexual organs, hygiene, and cleanliness etc. must be discussed. Health education is necessary to remove superstitions in the community. P1, G3: How can a building stand if the foundation is not strong?
			Health check-up camps	P7, G3: Health check-up camps should be organized at the village level. Information about pre-pregnancy care should be given through *Gram Sabhas* by *Gram Panchayat* members, especially female members. P4, G3: Women should be gathered together for group discussions by ASHAs at the village level.
	Community awareness	Empower younger women	Autonomy for planning pregnancy	P5, G3: Women should be educated so that they can take the decision whether she herself and the couple want a baby or delay the pregnancy.
			Demand generation	P7, G1: Women should be counseled about the need of planning pregnancy and being healthy before conceiving
			Support decision making	P6, G2: Women should be given information about pre-pregnancy services, and she should be provoked to think about her opinion and decision to plan pregnancy.
		Health education for older women	Delaying early marriages and adolescent pregnancies	P9, G4: It is important to have family support. The woman is certainly a member of the family and should be treated as such; others should take her care. P8, G1: Counseling should be done at this time (before conception) for taking mutual responsibility for a child, diet, BMI testing, etc. P9, G1: It is necessary to talk about the contraceptives' benefits to the couple and the mother-in-law as she plays an important role in decision making. P4, G1: It is important to talk with family, especially mother-in-law and husband. Everyone wants a child immediately after marriage.
			Reducing myths and misconceptions	P5, G2: The community should be informed about their wrong beliefs about services, food habits etc. There are local vegetables, fish, or crabs available in rivers, which are rich sources of vitamins and minerals. The use of these food items should be promoted.
		Male involvement	Involving male in planning pregnancy	P3, G4: Even the men should be educated regarding preconception care and involved in planning pregnancy. P2, G4: This education should be given in schools only, to create awareness among both girls and boys.

*WIFS, Weekly Iron and Folic acid Supplementation; ANM, Auxiliary Nurse Midwife; AWW, Anganwadi Worker; ASHA, Accredited Social Health Activist; ANC, Ante-natal Care; IUCD, Intra-uterine Contraceptive Devices; PHC, Primary Health Center; RH, Rural Hospital; BMI, Body Mass Index*.

### Individual

#### Knowledge of healthcare workers

Most of the participants had some knowledge about PCC and highlighted the importance of women's health before conception. Some key health aspects like physical and mental growth and development, hemoglobin levels, body mass index (BMI), illness, and medical history of a woman before pregnancy were narrated by HCW as important components of preconception services. Participants also reported the link between the ill health of the woman with low birth weight and adverse pregnancy outcomes. HCW were aware that there is no formal PCC program in the country. However, adolescent health program and family planning services were highlighted as the essential components of PCC services.

### Interpersonal

#### Work environment

Most of the participants informed about the heavy workload and workplace stress. They elaborated that compliance with supervisors' work pressure related to timely reporting took maximum time. This ultimately affected their primary work of providing services to the community. Few participants informed about poor support and co-operation from colleagues. The discrepancies in the payment of regular and contractual staff, and lack of appreciation and motivation from seniors were some of the points mentioned by participants for being dissatisfied.

### Community and social

#### Socio-economic

Frontline workers stated that poverty was one of the factors that aggravated women's health conditions. These women lack access to health services.

Participants reported a significant migration, especially among the newly married couples for earning opportunities. The migrated couple rarely avail health services at their workplace. The women are already in the second trimester when they return home, and it is too late to optimize her health status.

#### Socio-cultural norms and practices

##### Knowledge among community members

Participants reported that early marriages are common in the community. School drop-out rate are high among young girls as families prefer them to get married leaving schools. Despite their young age, married girls are pressured to conceive immediately after marriage. Participants observed insufficient knowledge, minimum self-awareness, and lesser autonomy in decision-making among women regarding planning a pregnancy or the use of family planning services. HCW also stated that after first delivery, many women may get Copper-T inserted in a government hospital but remove it in a private hospital within a few months and conceive. This short inter-pregnancy interval may lead to anemia, undernourishment among women, and adverse pregnancy outcomes.

HCW stated that preconception period was not viewed as a critical phase in the woman's reproductive cycle. Families would pay attention only when the pregnancy was confirmed. Very few women consulted health workers to avail preconception care services. Women would rarely think of eating balanced diet before pregnancy. The priority of serving food has always been given to family members than women themselves.

##### Substance abuse

*Mishri* (application of roasted tobacco on gums and teeth) and alcohol consumption were predominantly seen among older tribal women. The respondents mentioned that on coming across women consuming *Mishri* or alcohol, they tried to convince them to abstain from the habits, informing the side effects on their health and the health of the upcoming baby. However, it was usually not appreciated by women and their husbands.

##### Influence of older women

The community workers noted that the older woman in the household (mainly mother-in-law) usually influenced the decision to plan pregnancy. They invariably force the newly married women to conceive immediately after marriage (usually within 1 year of marriage). Many older women opposed spacing, arguing that they had undergone many pregnancies and deliveries without any complications. Convincing older women of the household about spacing was a significant challenge faced by health workers as these older women boast that they knew everything.

Health workers stated that the family members want at least two children (invariably including one son) at the earliest. After that, couples may opt for sterilization (mainly women). However, if the woman has one or more girls, she is almost compelled to conceive and has no say in planning her pregnancy. The family members do not allow women to decide for herself. Besides, the older women believe that it is the daughter-in-law who is responsible for any reproductive issues, including infertility. If she fails to conceive or cannot give birth to a son, the family members deem it her failure and may subject her to physical or mental violence.

##### Role of men

At times, married boys, in adolescent age group could not take decision regarding conception. Health workers reported low contraceptive use among married male partners irrespective of age. Older members of the household do not even accept contraceptive use by young, newly married men.

##### Myths and misconceptions

Participants perceived many myths and taboos regarding food habits and medication in the community. The community followed the tradition of not disclosing the pregnancy during the first trimester. Few older women do not allow younger newly married women to eat papaya, banana, horse gram (*Chana*), etc. They believe that it causes miscarriage/abortions. The taboo also exists regarding the consumption of Iron and Folic Acid (IFA) tablets, believing it leads to healthier, bigger-sized baby and thereby leading to complications during delivery. Women are not allowed to undergo any health check-ups in the pre-pregnancy stage, especially by older women. There is a lack of knowledge regarding Reproductive Tract Infections (RTI) or Sexually Transmitted Diseases (STD) among women. Women sometimes sought services from traditional healers for primary or secondary infertility or abortion.

The community members have misconceptions about government services also. People do not appreciate and avail government services, thinking them as substandard. Medications like Iron and Folic acid tablets, deworming tables, etc., are given free of cost but not consumed by the women.

### Organizational

#### Resources

Most participants emphasized the scarcity of human resources and specialized services at PHC. Further, health providers also highlighted scarcity of logistics, including irregular supply of investigation kits, medicines, and contraceptives, as one of the primary reasons leading to difficulties in diagnosis and treatment of acute or chronic conditions during preconception.

#### Health services

Participants highlighted adolescent health program and family planning services as the key services of PCC.

##### Adolescent health program

While discussing PCC, most participants spontaneously referred to the adolescent health program including provision of iron, folic acid and deworming tablets etc. The program is perceived as successful for adolescent school girls, but providing services to school drop-outs and migrants is challenging.

##### Family planning and other services

Family planning services are rarely provided to newly married couples. Medical services are provided in case of the irregular menstrual cycle or any other reproductive health-related problem or any diagnosed conditions. The participants often referred to antenatal and perinatal care. Participants explained that newly married women are regularly counseled about medical check-ups, but their compliance is poor. Women with chronic conditions are referred to secondary or tertiary health care centers. Nevertheless, these problems often remain unresolved due to inaccessibility, lack of proper services, or lack of confidentiality at the health care center. The issue of providing incentives to women was also raised. Incentives are often provided to the beneficiary for family planning and maternal health services. Frontline workers also drew attention to the ill effects of monetary incentives.

### Suggestions for implementation

#### Strengthening health system

HCW expressed the need for afresh guidelines for preconception care. Increasing the trained workforce and capacity building of human resources was suggested. The participants specially underlined the need of training and refreshers training to provide preconception care services. Most participants suggested that reducing workload, re-distribution of work, and minimizing discrepancies in the payments will help HCW work efficiently. They strongly conveyed the need of support and co-operation from all colleagues. Accredited Social Health Activists (ASHA) and Anganwadi workers (AWW) have a lesser population to cater to, leading to better reach in the community, and hence enhanced support from ASHAs and AWW is expected. Some of the participants suggested incentive-based utilization of ASHA's services for PCC. The need for specialist services at PHC level was insisted upon. They believed that the availability of specialist doctors at PHC would play a significant role in the early diagnosis and treatment of high-risk women in the preconception period. This would further help build trust in the community and affect the accessibility of health care services. Strengthening health care institutions in the delivery of PCC services through regular supplies of diagnostic kits and medicines was suggested. The need for a robust referral system was also insisted upon.

#### Special PCC program

Most of the participants emphasized that the services should begin with adolescents. The Weekly Iron and Folic acid supplementation (WIFS) program can be subsumed as a part of PCC. The participants also emphasized taking a detailed medical history, medical check-ups, and laboratory investigations before pregnancy. They insisted on testing sickle cell anemia as the district belongs to the highly prevalent belt. Besides the management of highly prevalent diseases like sickle cell anemia or chronic conditions like diabetes and hypertension; regular health check-up camps should be organized for diagnosis and treatment of reproductive tract infections.

#### Community awareness

##### Empower younger women

Participants thought that women's education plays a vital role in preconception care. Younger women can be empowered through health education, providing autonomy for planning pregnancy and generating demand for PCC services. Participants narrated that few educated women are less hesitant to come forward and ask about preconception care, use of contraceptives etc.

##### Health education for older women

Counseling was one of the essential strategies communicated by the respondents. It was suggested that families, especially the older women (mother-in-law), should be counseled, rather than individuals. Counseling of the entire family should be done before marriage and after marriage to include aspects like appropriate age of marriage, delaying pregnancy, increasing inter-pregnancy interval, the importance of PCC and health check-up before pregnancy, and prevention of domestic violence. Awareness about consuming a balanced diet, the need for three meals a day, the use of locally or seasonally available vegetables, and non-vegetarian items, especially seafood, should be encouraged.

Creating awareness in the community to dispel myths and taboos through health education was also recommended. Consumption of folic acid three months before conception to prevent neural tube defects should be informed on priority. Prevention of consanguineous marriage or between carriers of the sickle cell trait should also be included in counseling.

##### Male involvement

Awareness should also be created among male members to plan pregnancy. The use of contraceptives for delaying pregnancy, especially among adolescents, should be promoted.

## Discussion

FGD method involving 8–14 participants is well documented to understand the knowledge and explore the perceptions, attitudes, and suggestions about a specific topic through an active interaction (44). All the participants were frontline health workers; hence authors thought documenting their awareness and perceptions is important. All the FGDs were conducted by qualified and experienced persons, allowing the health workers to express their opinions freely in the absence of supervisors. This study provides evidence on the perceptions of the HCW in rural and tribal settings. The study observed no difference in perception of challenges and strategies between tribal and non-tribal areas. This may be due to the health workers' comparable qualifications and experience, regularly transferred from tribal to non-tribal areas and vice versa.

The SEM considers the complex interplay between individual, interpersonal, community, and institutional factors (43). The most common perceived barriers at the individual, interpersonal, and organizational levels were sub-optimal knowledge, heavy workload, stress, and inadequate health workforce and resources. The most common perceived barriers at the community and social level were insufficient knowledge about preconception care among women and the community and the influence of older women. These were inter-linked with other socio-economic criteria and socio-cultural norms and practices.

### Individual

Most of the HCW in this study have limited knowledge about PCC, which is similar to those observed in studies conducted in High-Income Countries like UK (31), and Low-Income Countries like Ethiopia (37, 38).

### Interpersonal and organizational

The perceived challenges in the present study included limited health workforce, availability of specialist services, challenges related to payments of health workers, heavy workload, stress and competing for work priorities, lack of comprehensive PCC program analogous with findings of other studies (32, 34). General Practitioners also observe payment differences and unclear allocation of responsibilities in developed countries (29, 45). Chuang et al. stated that though health providers are aware of the importance of positive health before pregnancy, preconception counseling is rarely initiated and is not a high priority for them (34).

### Community and social

The health care providers, mainly from High-Income Countries, report a similar lack of women's reproductive health knowledge, including PCC (32, 33, 35, 40, 45–47). This may be mainly due to the unawareness of the importance of preconception care in the community. A qualitative study in Pennsylvania observed that Physicians perceive rural community practices of unintended pregnancies, early childbearing, and large families as the major barriers to contraceptive and preconception care. They also added the absence of educational goals for career and life planning, including family planning, among younger women as the critical identified barriers (34). Another study by Peterson-Burch among the Latino population reports that families were reluctant to discuss reproductive health problems, particularly using birth control measures (30).

### Indian scenario

The challenges to the implementation of PCC like illiteracy of adult Indian women (28.5%), marriage before legal age (23.3%), adolescent pregnancy (6.8%), anemia in non-pregnant women (57.2%), unmet need for family planning and spacing (9.4% and 4.0% respectively), are still highly prevalent. Some health workers talk about family planning (23.9%) as reported in the National Family Health Survey-5 (NFHS) (48). The scenario is similar for the Nashik district (48). In India, many girls marry in adolescent age, have lower education and limited knowledge about reproductive health (49), lower decision-making autonomy (32, 50). It is also observed that mother-in-law influences the newly married woman's family planning decision (51, 52). Compliance with IFA supplementation during the peri-conception period varies from 5 to 43% (16, 37, 48). This low acceptance may be due to lack of knowledge, general feeling among women that conception and delivery are natural events and do not need intervention (53). Male involvement is low due to poor knowledge and gender norms (54).

### Model for PCC

Similar to the present study findings, capacity building and training of health staff and formulating guidelines for comprehensive PCC services are recommended by many studies (28, 30, 32, 40). In line with our study, other studies also emphasize creating awareness in the community about the need and availability of PCC services, which could be achieved through focused mass educational campaigns (28, 40). Promoting motivation and demand for informed choices about conception and birth spacing should be executed through counseling and women empowerment (32, 40). One Australian study proposed the availability of a checklist, patient brochures, hand-outs, and posters to support the delivery of PCC interventions (32).

**Figure 2:**
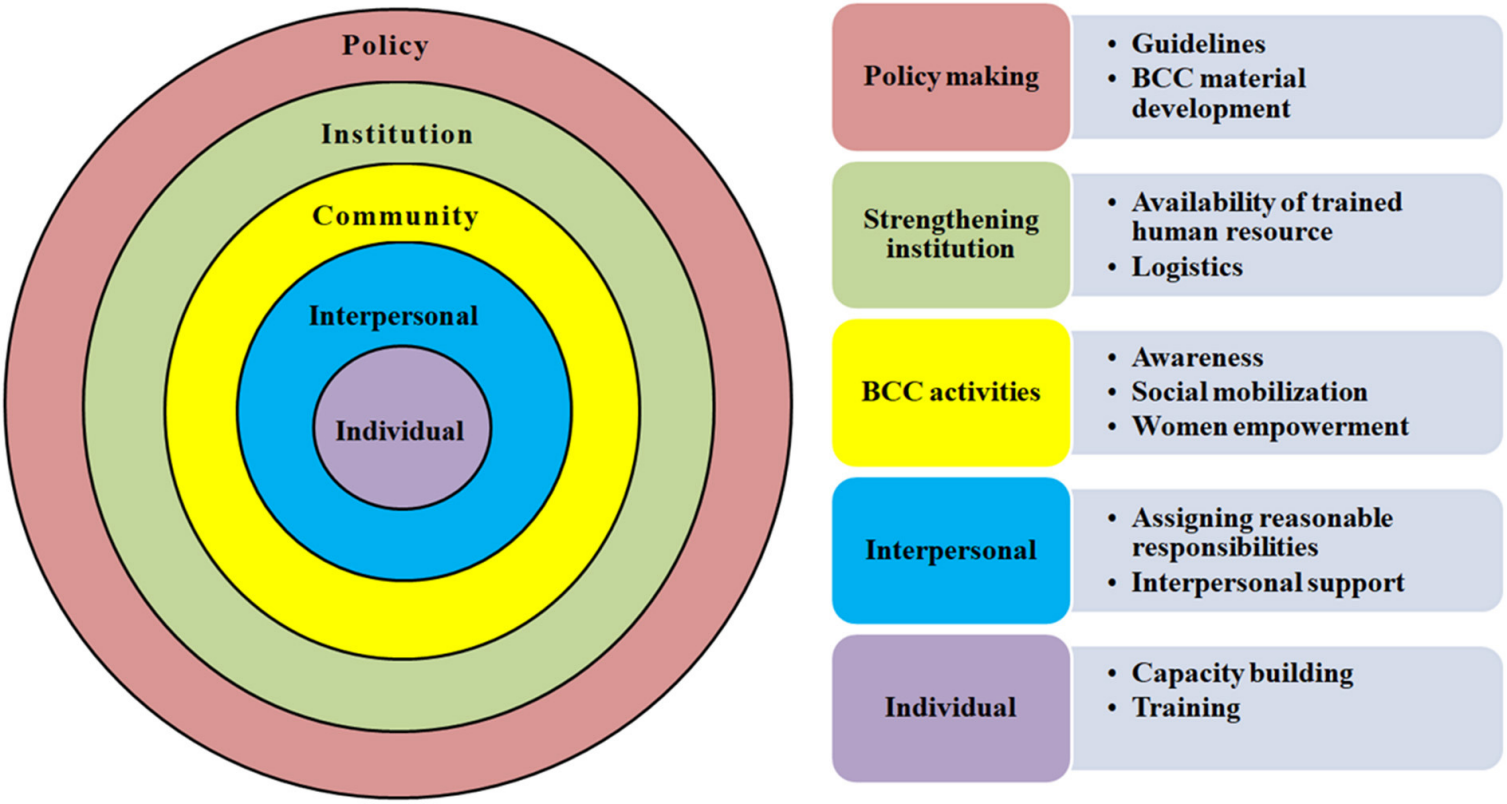
Conceptual framework of a model for preconception care services.

Thus, based on current findings, a model can be proposed for PCC services. Figure 2 depicts the core contents of this conceptual model based on the SEM framework, where the individual level shows minimal scope while the policy level shows the highest scope.

The model includes the dissemination of specific guidelines, health education material, and training of health workers. Behavioral Change and Communication (BCC) activities and material using different media can be developed and used during special awareness drives. The educational material should focus on demand generation, women empowerment, and social mobilization. Both, individual and group approaches (like *Gram Sabhas*) can be used for counseling. PCC may also be included as a part of *Pradhan Mantri Matru Vandana Yojana* (which provides specialized ANC services at PHC level on a specific day). This scheme should be supported with preconception care services, including medical examination and treatment if needed through capacity building of the healthcare workers, assigning reasonable responsibilities, and interpersonal support. These interventions will bring sustainable behavioral change to the community.

The situation and the challenges in most LMICs are similar to those identified in the present study. Hence the proposed model for PCC may be used as a preliminary guide by the LMICs for developing and implementing the PCC interventions. Further research should continue to determine the effect of this model in delivering PCC.

### Strengths and limitations

The unique feature is that the study provides insights into the perceptions of the frontline health workers in rural settings (including tribal), considering multi-level factors of the SEM influencing the PCC services. The small sample size involving only one district limits the generalizability of the findings. The absence of representation from the urban and private sectors is another limitation. After a few initial meetings about the larger intervention project, the authors conducted this study. Hence, there is a possibility of acquiring some knowledge by HCW at PHC or subcenter before focus group discussions. Despite these limitations, study findings helped to develop a model strategy for implementing PCC services in the primary health system.

## Conclusion

The study identifies key challenges perceived by the frontline healthcare workers as inadequate workforce and lack of knowledge and acceptance about preconception care services in the community, especially older female family members. Furthermore, it provides a model framework that can be used to develop strategies for implementing preconception care services.

We recommend an urgent need of institutional strengthening and human resource capacity building for a comprehensive PCC program. Appropriate BCC material designing for community awareness about PCC would be one of the best strategies for implementing PCC in the health care delivery system. The government may introduce PCC to address the gap in the continuum of care and give due importance. PCC can be an integral component of reproductive and child health, and the concerned program can be renamed Reproductive, Preconception, Maternal Neonatal Child Health and Adolescents (RPMNCHA).

## Data availability statement

The raw data supporting the conclusions of this article will be made available by the authors, on reasonable request.

## Ethics statement

The study was approved by the Institutional Ethics Committee (DCGI Regd. No. ECR/518/Inst/MH/2014/RR-17) vide letter number BVDUMC/IEC/11 dated 30th April 2018. Written informed consent for participation, and audio recording was obtained from all participants prior to the focus group discussion.

## Author contributions

AC, JG, PP, and SP designed and conducted interviews under the supervision of PD. MK and AD helped in the recruitment of participants. AC, PD, and JG independently identified the themes, sub-themes, and conducted data analysis. AC, PD, JG, PP, and SP discussed and finalized the themes and sub-themes. AC prepared the initial draft of the manuscript. PD, JG, and PP undertook a critical review of the manuscript. AP, AS, PD, KB, AD, and AC drafted the guideline for the comprehensive public health interventions and its assessment. All authors conceived the study, reviewed, and approved the published version of the manuscript.

## Funding

This research was funded by UNICEF, Maharashtra, India through Department of Public Health, Government of Maharashtra.

## Conflict of interest

The authors declare that the research was conducted in the absence of any commercial or financial relationships that could be construed as a potential conflict of interest.

## Publisher's note

All claims expressed in this article are solely those of the authors and do not necessarily represent those of their affiliated organizations, or those of the publisher, the editors and the reviewers. Any product that may be evaluated in this article, or claim that may be made by its manufacturer, is not guaranteed or endorsed by the publisher.
