# Supplementary material for: Perceptions of and challenges faced by primary healthcare workers about preconception services in rural India: A qualitative study using focus group discussion
Source: Front Public Health. 2022 Aug 17;10:888708. doi: 10.3389/fpubh.2022.888708 (PMC9432348; doi:10.3389/fpubh.2022.888708)
Supplement: Supplementary file 1 [file Table_1.doc]

| **S1: Interview guide for FGD of health care providers** | | |
| --- | --- | --- |
| **Domain area** | **Sub-domain area** | **Probes** |
| Knowledge about preconception care | Awareness | Have you ever heard about preconception care? |
|  | Content | What have you heard about preconception care? |
|  | Necessity | Do you think, preconception care is necessary in health care system? Express your opinion regarding preconception care in relevance to safe mother and healthy baby |
| Existing health care services for preconception care | PCC services provided | What services do you provide for preconception care? |
|  | Community views | What are the views of community about preconception care? |
|  | Community demand | Is there any demand for knowledge and/or services for preconception care from the community? |
| Challenges/ gaps/ barrier in providing preconception services | Challenges from provider side | Are there any challenges at your level? What are they?  Are there any challenges at institutional level? What are they? |
|  | Challenges from community side | Are there any challenges at community level? What are they? |
| Probable readdress mechanisms | Strategies to address provider side challenges | Can provider side challenges be addressed?  What solutions do you suggest for these challenges/ gaps that you mentioned? |
|  | Strategies to address community side challenges | Can community side challenges be addressed?  What solutions do you suggest for these challenges/ gaps that you mentioned? |
| Strategies for implementing PCC | Content | What should be included in preconception care? |
|  | Primary service provider | Who should primarily provide preconception care services? |
|  | Your role | What role would you like to perform in providing these services? |
|  | Suggestions | What are your suggestions to improve the health of mother and baby? |
| Summarize: Key messages of the FGD and discussion | | |
